# Case report: Relapsed/refractory extranodal natural killer/T-cell lymphoma nasal type with extensive central nervous system involvement

**DOI:** 10.3389/pore.2022.1610866

**Published:** 2023-01-09

**Authors:** Austėja Dapkevičiūtė-Purlienė, Vytautas Augustinavičius, Andrius Žučenka

**Affiliations:** ^1^ Faculty of Medicine, Institute of Clinical Medicine, Vilnius University, Vilnius, Lithuania; ^2^ Department of Radiology, Nuclear Medicine and Physics of Medicine, Faculty of Medicine, Center for Radiology and Nuclear Medicine, Vilnius University, Vilnius, Lithuania

**Keywords:** case report, lymphoma, NK-cell, T-cell, central nervous system

## Abstract

**Background:** Extranodal natural killer/T-cell lymphoma (ENKL) is a rare subtype of mature T and natural killer cell lymphomas associated with Epstein-Barr virus.

**Case:** A 20-year-old presented with severe neurological symptoms and was diagnosed with stage IV ENKL, nasal type, with CNS involvement. Overall, the patient received nine treatment lines, including chemotherapy, craniospinal irradiation, allogeneic stem cell transplant (alloSCT), donor lymphocyte infusions, and novel agents (Nivolumab, Daratumumab, Thalidomide, Lenalidomide, virus-specific T cells) combined with intrathecal chemotherapy. The treatment effect was evaluated in both blood and CSF (cerebrospinal fluid). First-line SMILE chemotherapy resulted in systemic and CNS remission. Later Cytarabine-based chemotherapy and Daratumumab combination helped to reinduce remission before alloSCT.

**Conclusion:** We show that efficacy monitoring should include both blood and CSF analysis. High-dose Cytarabine-based chemotherapy in combination with Daratumumab and intrathecal chemotherapy may be considered as salvage CNS-directed therapies. We add to existing limited data that Daratumumab penetrates the blood-brain barrier.

## Introduction

Extranodal natural killer/T-cell lymphoma (ENKL), nasal type, is a rare subtype of mature T and natural killer cell lymphomas which is associated with Epstein-Barr virus (EBV) (1). ENKL comprises only 1% of all lymphomas in Western countries (2), and only 3% of all ENKL cases are associated with central nervous system (CNS) involvement (3).

Due to the expression of P-Glycoprotein by ENKL cells, non-anthracycline based chemotherapy in combination with radiotherapy remains the standard of care, however, subsequent treatment lines are poorly described (1). Even less data are available regarding therapeutic approaches when extensive CNS involvement is present due to variable ability to cross the blood-brain barrier (BBB). Therefore, relapsed or refractory (R/R) ENKL involving CNS usually leads to an unsatisfactory prognosis (1). Herein we describe a case of highly resistant ENKL with extensive CNS involvement treated with nine different treatment lines including chemotherapy, radiotherapy, allogeneic stem cell transplantation (alloSCT), and multiple novel agents. In addition, we demonstrate the effect of these therapies on both systemic and CNS disease and provide new insights regarding the treatment of ENKL with CNS involvement.

## Case report

A previously healthy 20-year-old Caucasian female presented with headache, nausea, vomiting, plegia of both legs and left arm, urinary and faecal retention, partial ophthalmoplegia, and hearing impairment in April 2019. The symptoms had gradually worsened over 2 months.

**FIGURE 1:**
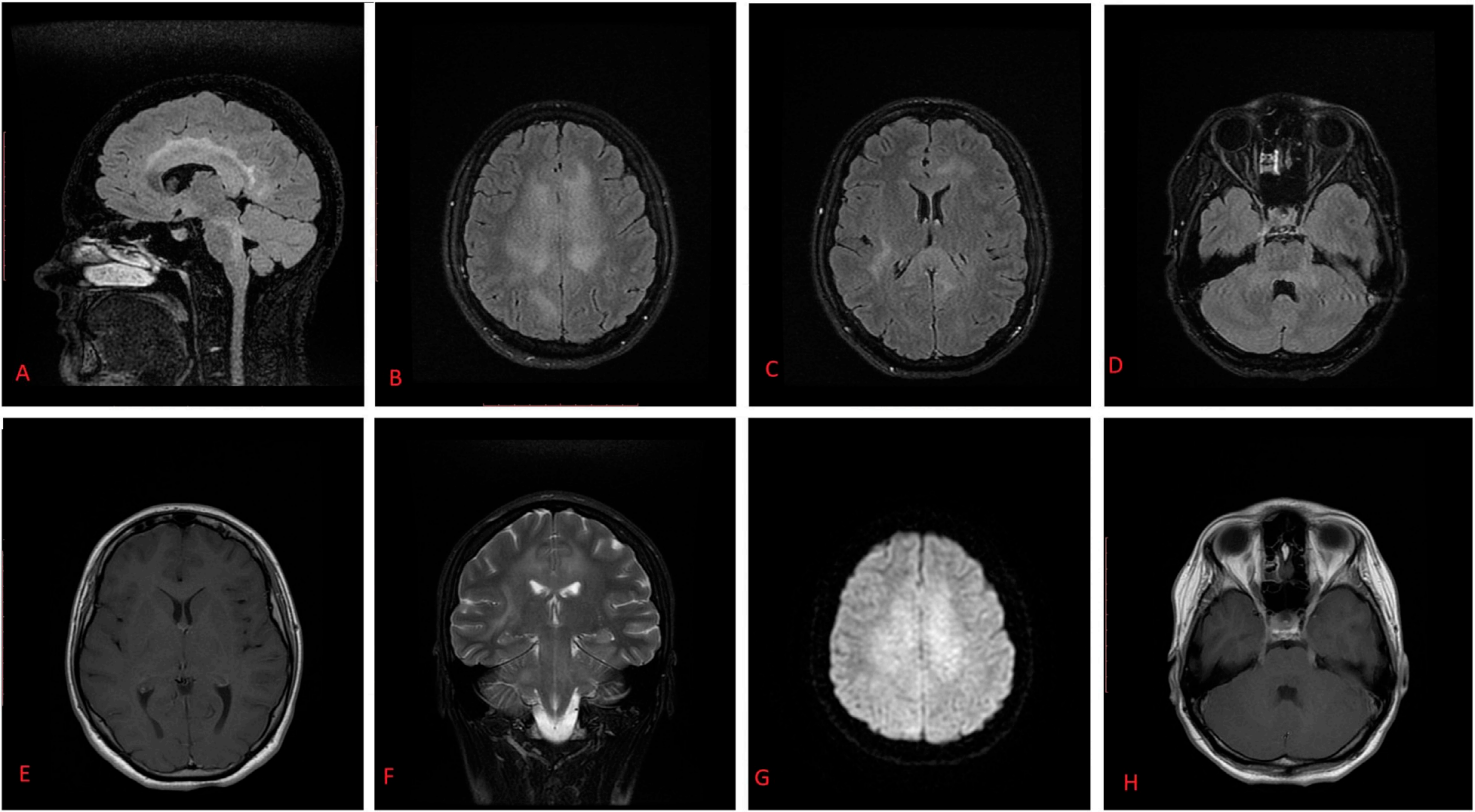
T2 FLAIR MR images show hyperintense right corticospinal tract **(A)**, corona radiata, frontal and parietal lobes signal **(B)**, corpus callosum dorsal parts **(C)**, cerebral peduncular, and hemisphere white matter **(D,F)**. Contrast-enhanced T1-weighted axial MR image shows no visible contrast enhancement or intraaxial changes **(E)**. Diffusion-weighted axial MR images show slightly restricted diffusion **(G)**. Contrast-enhanced T1-weighted axial MR images show enlargement of cranial nerves bilaterally (n.oculomotorius, and n. trigeminus) **(H)**. The differential of these MR findings included viral encephalitis, demyelinating disease, lymphoma, and leptomeningeal metastases.

Head MRI showed diffuse changes bilaterally (Figure 1). Lumbar puncture was done and showed 1,944,000 EBV copies/ml and increased cytosis of 2300 × 10^9^/l with 81% of abnormal lymphoid cells. Flow cytometry results (90% of aberrant cells in cerebrospinal fluid expressing CD45^+^, CD2^+^, CD8^−/+^, CD56^+^, CD38^+^, CD16^−/+^, CD94^+^, CD81^+^, HLA-DR+) and sinus biopsy were consistent with a diagnosis of stage IV ENKL, nasal type, with CNS involvement.

**FIGURE 2:**
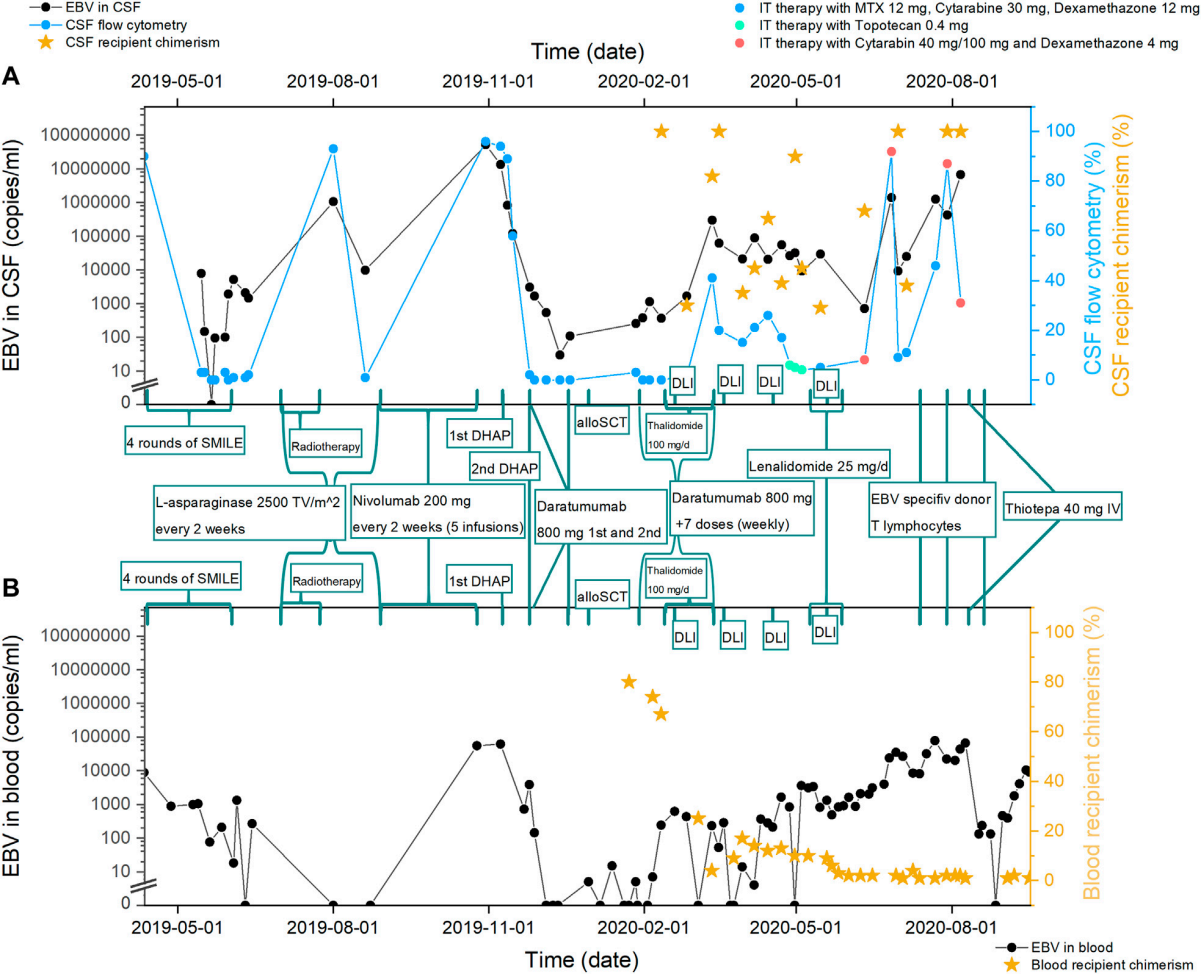
Treatment regimens given to the patient and treatment effect on: **(A)** CNS disease. Evaluated as—EBV copy number in CSF (cerebrospinal fluid), percent of ENKL cells in CNS flow cytometry, percent of recipient chimerism in CSF (after alloSCT). Every measurement point of CSF analysis was also intrathecal prophylaxis with chemotherapeutic agents. **(B)** Systemic disease. Evaluated as—EBV copy number in blood, percent of recipient chimerism in the blood (after alloSCT). SMILE, Etoposide, Ifosfamide, Methotrexate, Dexamethasone, and Peg-asparaginase; Radiotherapy, nasal and craniospinal irradiation; DHAP, dexamethasone, cytarabine and cisplatin; alloSCT, allogeneic stem cell transplantation; DLI, donor lymphocyte infusion; VST, virus-specific T cells; MRI, head magnetic resonance imaging.

**FIGURE 3:**
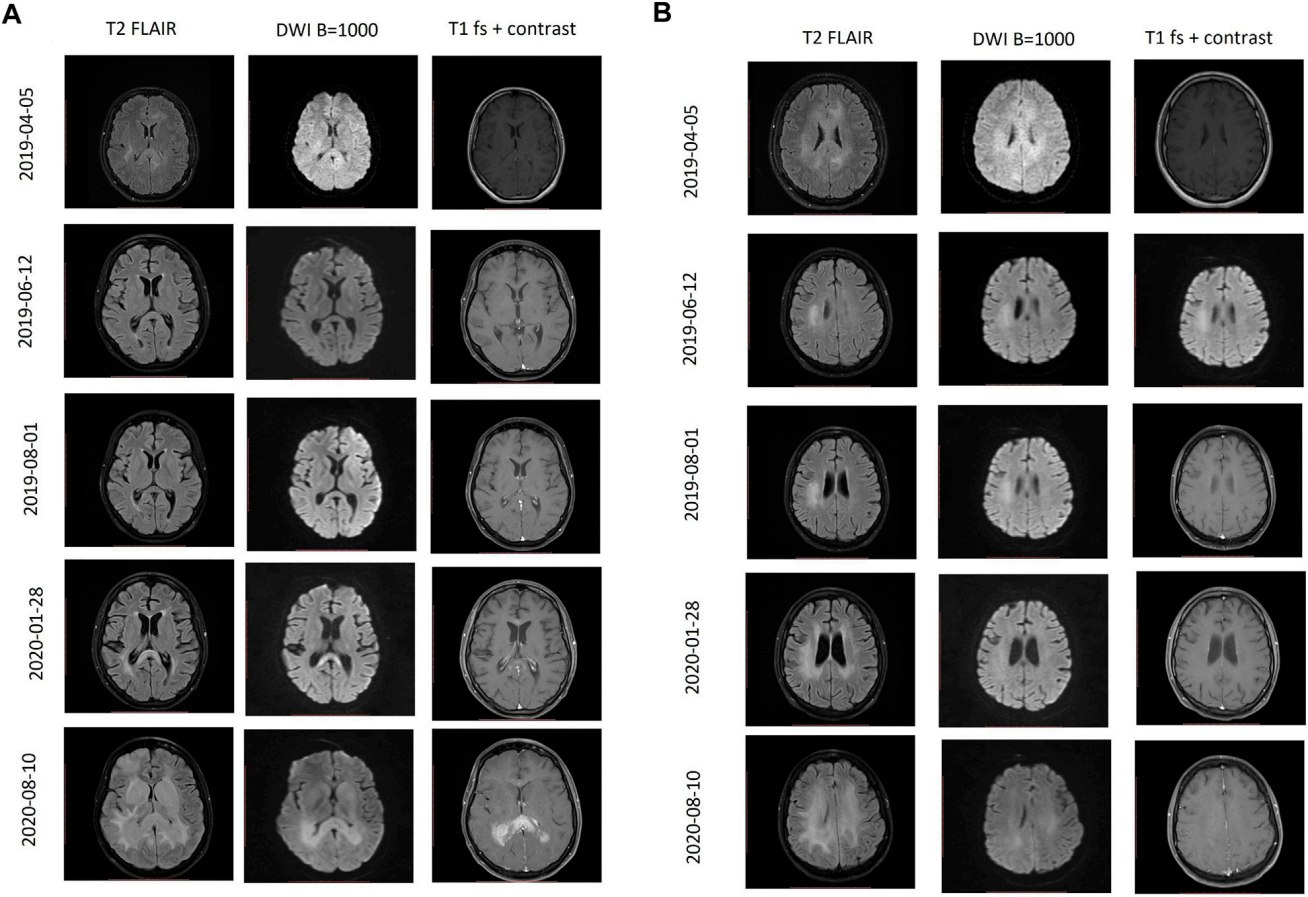
Follow-up MRI scans during different periods of treatment. **(A)** T2 FLAIR, DWI, contrast-enhanced T1 axial MR images at the level of basal ganglia. **(B)** T2 FLAIR, DWI, contrast-enhanced T1 axial MR images at the level of frontal horns of the lateral ventricles. 05 April 2019 Primary MRI scan. 12 June 2019 MRI follow-up examination after completion of first-line chemotherapy shows revisal of the hyperintensity lesions in the right parietal lobe, corpus callosum, and corona radiata. 01 August 2019 MRI follow-up examination demonstrates the progression of the lesion in the right parietal lobe. No additional lesions were found. 28 January 2020 MRI follow-up examination demonstrates enlarged enhancing lesion of the dorsal part of the corpus callosum with restricted diffusion. 10 August 2020 MRI follow-up examination demonstrates T2 hyperintense contrast-enhancing masses of the dorsal part of corpus callosum extending to basal ganglia with significantly restricted diffusion, perifocal edema, and compression of the ventricle system.

During the treatment period of 1.5 years, the patient received nine treatment lines, including SMILE chemotherapy with following nasal and craniospinal irradiation, alloSCT, and novel agents (Nivolumab, Daratumumab, Thalidomide, Lenalidomide, virus-specific T cells) combined with intrathecal chemotherapy as shown in Figure 2. After the first line of SMILE chemotherapy with concomitant intrathecal triplets, the patient’s neurological condition improved significantly, ophthalmoplegia disappeared (which resulted in regained vision), hearing improved, pelvic organ function normalized, she regained full control of both arms and started walking with some assistance. Changes in MRI images during various treatment lines can be seen in Figure 3. Neurological improvement, with some fluctuation, lasted until the last month before death due to disease progression in October 2020.

## Discussion

We report an extremely rare case of ENKL with CNS involvement as the main presentation. Despite the poor outcome, our efforts resulted in significantly improved neurological condition and quality of life for 16 months.

Regarding the previously reported CNS ENKL outcomes, survival of 1.5 years from the diagnosis is one of the best published results. Previous cases and small studies have shown survival of only 3–6 months when ENKL presents with CNS involvement (3, 4). These reports used chemotherapy as the main treatment approach.

Our case is unique due to the rarity of the disease and numerous treatment lines used. This case is also the first one directly comparing different treatment effects on systemic versus CNS disease.

### Monitoring treatment efficacy

It is well known that ENKL is associated with EBV copy number in blood; therefore, it can be used to evaluate treatment efficacy (1, 2, 5). We add that EBV CSF counts correlate well with CSF flow cytometry and may be used for CNS ENKL disease monitoring (Figure 2). To the best of our knowledge, the use of CSF EBV counts for disease monitoring has not been described before. In addition, CSF chimerism after alloSCT is another useful marker, previously described only for different diagnostic entities (6, 7).

### First-line treatment

Currently, it is thought that the best possible first-line treatment for ENKL is a combination of non-anthracycline based chemotherapy and radiotherapy (1). The highest response rates are seen with SMILE (Dexamethasone, methotrexate, ifosfamide, L-asparaginase or pegasparaginase, and etoposide) and a less toxic GDP (Gemcitabine, Dexamethasone, Cisplatin) regimen (1, 5). We chose SMILE because methotrexate penetrates BBB well, while agents included in GDP do not. First-line therapy significantly improved neurological condition and cleared EBV in both CSF and blood. During the consolidation with craniospinal irradiation, blood EBV remained undetectable, however, CSF EBV peaked with concomitant radiological progression, establishing the R/R CNS ENKL diagnosis.

### Management of R/R ENKL

Options are scarce and ill-defined in R/R ENKL, especially with CNS involvement.

#### Nivolumab

Programmed cell death ligand 1 (PD-L1) is expressed in >80% of ENKL patients; therefore, immune checkpoint inhibitors may be a possible treatment option for R/R ENKL (8). In one study, low-dose Nivolumab (40 mg every 2 weeks) was used in three patients with R/R ENKL. Two patients responded, but the last patient with CNS involvement achieved only radiological disease stabilization (9). A few small studies demonstrate the efficacy of Pembrolizumab in the R/R ENKL with 11/14 patients achieving CR/PR (10, 11) Existing literature demonstrates that Nivolumab crosses BBB (12–14). We administered standard Nivolumab doses (200 mg every 2 weeks), which has not been previously described in ENKL (9). Treatment was well tolerated, however, there was no response in either systemic or CNS disease (Figure 2). This is possibly due to the lack of PD-1/PDL-1 expression in diagnostic samples. The relation between the expression of PD1-ligand and clinical response remains unclear to date (10).

#### Daratumumab

The rationale to use anti-CD38 antibody Daratumumab is based on the expression of CD38 in most ENKL cases. Several case reports and a small phase 2 study demonstrated some efficacy of Daratumumab in R/R ENKL (2, 15). Our patient received Daratumumab in combination with DHAP and intrathecal chemotherapy, which resulted in a good response before alloSCT. It remains unclear if Daratumumab crosses the BBB since CNS involvement was an exclusion criterion in the registration studies (16). Interestingly, our patient lost CD38 expression in the CSF after several Daratumumab infusions, possibly indicating the ability of Daratumumab to cross the BBB. This finding had also been reported in a single multiple myeloma patient (16). After the loss of CD38, Daratumumab became ineffective in controlling the disease (Figure 2). Overall, Daratumumab either alone or in combination with systemic chemotherapy may be used for salvage therapy even if CNS is the main affected site in ENKL, though monitoring of the CD38 expression is recommended.

#### DHAP

Cisplatin/Carboplatin containing regimens (GDP, ESHAP, DeVIC, etc.) are usually recommended for treating R/R ENKL (1). We chose the less-used DHAP (Cytarabine, Cisplatin, and Dexamethasone) regimen due to the ability of high-dose Cytarabine to penetrate into the CNS (17). The combination of the backbone DHAP with intrathecal triplets and Daratumumab resulted in the total elimination of ENKL cells in the CSF (flow cytometry), blood EBV clearance, and one of the lowest EBV counts in the CSF throughout the whole course of the disease. This may indicate the efficacy of Cytarabine in the R/R CNS ENKL setting.

#### Immunomodulators—Thalidomide and Lenalidomide

Immunomodulators deactivate the NF-κB pathogenetic pathway which is important in ENKL pathogenesis (18). One study showed that adding Thalidomide to chemotherapy may increase CR, PFS, and OS in T and NK lymphomas (19). A few case reports also show positive results achieved with Lenalidomide monotherapy (20, 21). Thalidomide and Lenalidomide are known to cross BBB based on previous primate experimental studies (22) and successful case reports with multiple myeloma involving CNS (16, 23).

Therefore, after an early relapse post alloSCT, we initiated Thalidomide in combination with donor lymphocyte infusions (DLI). Thalidomide was firstly chosen over Lenalidomide due to a lower risk of GVHD (graft-versus-host disease) and lower risk of cytopenias. However, Thalidomide was discontinued after 3 weeks due to the increasing EBV counts. Two months later we added Lenalidomide. Despite the decrease of the EBV counts, Lenalidomide was stopped after 3 weeks due to the rapid onset of Grade 4 acute gastrointestinal and liver GVHD. Overall, immunomodulators may have a role in CNS ENKL. While Lenalidomide has stronger immunomodulatory properties than Thalidomide, the higher risk of myelosuppression and GVHD in the post alloSCT setting must be considered.

#### autoSCT/alloSCT

Current guidelines favour the use of autologous SCT as front-line consolidation therapy for disseminated ENKL, whereas alloSCT is mainly performed in the R/R ENKL setting providing long-term remission in some patients (1). At the time of alloSCT our patient had R/R disease and available matched related donor, therefore, we performed an alloSCT using reduced intensity conditioning with Fludarabine/Cyclophosphamide/ATG + high-dose Cytarabine. It is debatable whether the myeloablative conditioning with CNS penetrating agents Busulphan and Thiotepa would have eradicated the residual CNS disease, but the patient’s condition was unsuitable for a more intensive conditioning regimen at that moment.

#### VST

EBV-associated malignancies usually express latent membrane proteins (LMPs) that are targeted by VST (virus-specific T cells). Several small studies have reported durable ENKL remissions after VST therapy, nevertheless, none of the patients had CNS involvement (1). McLaughin et al. (24) suggest the rationale to VST as maintenance rather than an active therapy for relapsed disease. The efficacy of VST as a CNS-directed therapy remains unknown, as only a few case reports demonstrated positive results in patients with progressive multifocal encephalopathy (25). Our patient received three VST infusions without significant response or toxicity. The lack of efficacy may be attributable to the imperfect VST penetration into CSF, or the fact that we used it for a relapsed disease instead of as a form of maintenance.

#### DLI

Efficacy data of systemic DLIs for CNS haematological malignancies are lacking (26). In our case, systemic DLIs had a fast positive effect on systemic disease, with barely any effect on CNS involvement (Figure 2). Another debatable option is the CNS-directed intrathecal DLIs. There are 7 case reports with various CNS relapses demonstrating some efficacy and acceptable toxicity (6, 7). In addition, three of these cases presented successful treatment of EBV-associated CNS post-transplant lymphoproliferative disorder resulting in the disappearance of all radiological lesions, EBV clearance, and clinical improvement (7). Due to the technical difficulties and the high risk of GVHD we did not use intrathecal DLIs. Nevertheless, this approach may be considered in the post-alloSCT patients in case of CNS relapse.

## Conclusion

ENKL with CNS involvement is a rare and aggressive disease. Limited data exist on the efficacy of various treatment entities especially with CNS disease in relapsed/refractory setting. To the best of our knowledge, this is the first case directly comparing nine different treatment lines (chemotherapy, radiotherapy, alloSCT, and multiple novel agents) efficacy on both systemic and CNS disease. We show that treatment efficacy monitoring should include CSF flow cytometry, EBV copy number, and donor/recipient chimerism (post alloSCT) in both blood and CSF. High-dose Cytarabine-based chemotherapy in combination with Daratumumab and intrathecal chemotherapy may be considered as salvage CNS-directed therapies. We add to existing limited data that Daratumumab penetrates the blood-brain barrier. More studies are needed to define the best approach in this rare, high-risk disease.

## Data Availability

The raw data supporting the conclusion of this article will be made available by the authors, without undue reservation.

## References

[1] Yamaguchi M Suzuki R Oguchi M. Advances in the treatment of extranodal NK/T-cell lymphoma, nasal type [Internet]. Blood. (2018) 131131(23):2528–40. 10.1182/blood-2017-12-791418 29602763

[2] Yamaguchi M Miyazaki K. Current treatment approaches for NK/T-cell lymphoma. J Clin Exp Hematop 57:98–108. 10.3960/jslrt.17018 PMC6144191 28679966

[3] Yang Y Li Z Zhiyang C Liang H. Extranodal natural killer/T-cell lymphoma nasal type with central nervous system involvement mimicked tuberculous meningitis: A case report. Medicine (Baltimore) 98(34):e16747. 10.1097/MD.0000000000016747 PMC6716710 31441847

[4] Romero-Guadarrama MB Aguilar-Martinez E. Extranodal nasal NK/T-cell lymphoma with dissemination to the central nervous system: A case report. Acta Cytol (2010) 54(5):993–7. 21053585

[5] Tse E Kwong YL. Diagnosis and management of extranodal NK/T cell lymphoma nasal type. Expert Rev Hematol 99(9):861–71. 10.1080/17474086.2016.1206465 27347812

[6] Yanagisawa R Nakazawa Y Sakashita K Saito S Tanaka M Shiohara M Intrathecal donor lymphocyte infusion for isolated leukemia relapse in the central nervous system following allogeneic stem cell transplantation: A case report and literature review. Int J Hematol 103:107–11. 10.1007/s12185-015-1902-1 26586462

[7] Zhao J Zu Y Han L Zhang Y Gui R Yu F Treatment of Epstein–Barr virus associated central nervous system diseases after allogeneic hematopoietic stem cell transplantation with intrathecal donor lymphocyte infusion. Bone Marrow Transpl (2019) 54(6):821–7. 10.1038/s41409-018-0409-9 30518982

[8] Yamaguchi M Oguchi M Suzuki R. Extranodal NK/T-cell lymphoma: Updates in biology and management strategies [Internet]. Best Pract Res Clin Haematol (2018) 3131(3):315–21. 10.1016/j.beha.2018.07.002 30213402

[9] Chan TSY Li J Loong F Khong PL Tse E Kwong YL. PD1 blockade with low-dose nivolumab in NK/T cell lymphoma failing l-asparaginase: Efficacy and safety [internet]. Ann Hematol 9797(1):193–6. 10.1007/s00277-017-3127-2 28879531

[10] Li X Cheng Y Zhang M Yan J Li L Fu X Activity of pembrolizumab in relapsed/refractory NK/T-cell lymphoma. J Hematol Oncol (2018) 11(1):15. 10.1186/s13045-018-0559-7 29386072 PMC5793390

[11] Kwong YL Chan TSY Tan D Kim SJ Poon LM Mow B PD1 blockade with pembrolizumab is highly effective in relapsed or refractory NK/T-cell lymphoma failing L-asparaginase. Blood. (2017) 129(17):2437–42. 10.1182/blood-2016-12-756841 28188133

[12] Abid H Watthanasuntorn K Shah O Gnanajothy R. Efficacy of pembrolizumab and nivolumab in crossing the blood brain barrier. Cureus (2019) 11(4):e4446. 10.7759/cureus.4446 31245230 PMC6559690

[13] Van Bussel MTJ Beijnen JH Brandsma D. Intracranial antitumor responses of nivolumab and ipilimumab: A pharmacodynamic and pharmacokinetic perspective, a scoping systematic review. BMC Cancer (2019) 19(1):519. 10.1186/s12885-019-5741-y 31146733 PMC6543612

[14] Fan S Ren H Zhao L Yin J Feng G Wang J Neurological immune-related adverse events associated with immune checkpoint inhibitors: A review of the literature. Asia Pac J Clin Oncol (2020) 16(6):291–8. 10.1111/ajco.13375 32893999

[15] Kim W-S Eom H-S Yeh S-P Cho S-G Heo DS Kim JS Daratumumab monotherapy for patients with relapsed or refractory (R/R) natural killer/T-cell lymphoma (NKTCL), nasal type: An open-label, single-arm, multicenter phase 2 study. Blood (2018) 132(1):1617. 10.1182/blood-2018-99-111885 PMC7885403 33588922

[16] Varga G Mikala G Gopcsa L Csukly Z Kollai S Balázs G Multiple myeloma of the central nervous system: 13 cases and review of the literature. J Oncol (2018) 2018:3970169. 10.1155/2018/3970169 29849629 PMC5937370

[17] Ferreri AJ Reni M Foppoli M Martelli M Pangalis GA Frezzato M High-dose cytarabine plus high-dose methotrexate versus high-dose methotrexate alone in patients with primary CNS lymphoma: A randomised phase 2 trial. Lancet (2009) 374(9700):1512–20. 10.1016/S0140-6736(09)61416-1 19767089

[18] De Mel S Hue SSS Jeyasekharan AD Chng WJ Ng SB. Molecular pathogenic pathways in extranodal NK/T cell lymphoma. J Hematol Oncol 12(1):33. 10.1186/s13045-019-0716-7 PMC6444858 30935402

[19] Wu H Zhao C Gu K Jiao Y Hao J Sun G. Thalidomide plus chemotherapy exhibit enhanced efficacy in the clinical treatment of T-cell non-hodgkin’s lymphoma: A prospective study of 46 cases. Mol Clin Oncol 2:695–700. 10.3892/mco.2014.307 25054032 PMC4106729

[20] Wang L Wang ZH Chen XQ Wang KF Huang HQ Xia ZJ. First-line combination of GELOX followed by radiation therapy for patients with stage IE/IIE ENKTL: An updated analysis with long-term follow-up. Oncol Lett 10(2):1036–40. 10.3892/ol.2015.3327 PMC4509369 26622621

[21] Kumar A Gupta P Kaushal V. Locally advanced angiocentric nk/t cell lymphoma of nasal cavity. J Cancer Prev Curr Res (2018) 9(6):00366. 10.15406/jcpcr.2018.09.00366

[22] Muscal JA Sun Y Nuchtern JG Dauser RC McGuffey LH Gibson BW Plasma and cerebrospinal fluid pharmacokinetics of thalidomide and lenalidomide in nonhuman primates. Cancer Chemother Pharmacol 69(4):943–7. 10.1007/s00280-011-1781-y PMC3685292 22109830

[23] Rubenstein JL Geng H Fraser EJ Formaker P Chen L Sharma J Phase 1 investigation of lenalidomide/rituximab plus outcomes of lenalidomide maintenance in relapsed CNS lymphoma. Blood Adv 2(13):1595–607. 10.1182/bloodadvances.2017014845 PMC6039666 29986852

[24] McLaughlin LP Rouce R Gottschalk S Torrano V Carrum G Wu MF EBV/LMP-specific T cells maintain remissions of T- and B-cell EBV lymphomas after allogeneic bone marrow transplantation. Blood 132(22):2351–61. 10.1182/blood-2018-07-863654 PMC6265652 30262660

[25] Muftuoglu M Olson A Marin D Ahmed S Mulanovich V Tummala S Allogeneic BK virus–specific T cells for progressive multifocal leukoencephalopathy. N Engl J Med 379(15):1443–51. 10.1056/NEJMoa1801540 PMC6283403 30304652

[26] Seo S Kami M Honda H Kashima T Matsumura T Moriya A Extramedullary relapse in the so-called “sanctuary” sites for chemotherapy after donor lymphocyte infusion. Bone Marrow Transplant (2000) 25(2):226–7. 10.1038/sj.bmt.1702116 10673689

